# Microbial Multidrug-Resistant Organism (MDRO) Mapping of Intensive Care Unit Infections

**DOI:** 10.3390/medicina61071220

**Published:** 2025-07-04

**Authors:** Ahmed Yassin, Ragaey Ahmad Eid, Mohammad Farouk Mohammad, Marwa O. Elgendy, Zeinab Mohammed, Mohamed E. A. Abdelrahim, Ahmed M. Abdel Hamied, Reem Binsuwaidan, Asmaa Saleh, Mona Hussein, Eman Hamdy Mohamed

**Affiliations:** 1 Department of Critical Care Medicine, Faculty of Medicine, Beni-Suef University, Beni-Suef 62514, Egypt; dr.ahmedelsisi@yahoo.com; 2 Department of Gastroenterology, Hepatology and Infectious Diseases, (Tropical Medicine Department), Faculty of Medicine, Beni-Suef University, Beni-Suef 62514, Egypt; 3 Department of Chest Diseases, Faculty of Medicine, Beni-Suef University, Beni-Suef 62514, Egypt; doctor.m79@gmail.com; 4 Department of Clinical Pharmacy, Beni-Suef University Hospitals, Faculty of Medicine, Beni-Suef University, Beni-Suef 62514, Egypt; 5 Department of Public Health and Community Medicine, Faculty of Medicine, Beni-Suef University, Beni-Suef 62514, Egypt; zynab.mohammed@med.bsu.edu.eg; 6 Clinical Pharmacy Department, Faculty of Pharmacy, Beni-Suef University, Beni-Suef 62521, Egypt; mohamed.abdelrahim@pharm.bsu.edu.eg; 7 Department of Pediatrics, Faculty of Medicine, Nahda University (NUB), Beni-Suef 62513, Egypt; ahmed.abdelhamid@nub.edu.eg; 8 Department of Pharmaceutical Sciences, College of Pharmacy, Princess Nourah bint Abdulrahman University, Riyadh 11671, Saudi Arabia; rabinsuwaidan@pnu.edu.sa (R.B.); asali@pnu.edu.sa (A.S.); 9 Department of Neurology, Faculty of Medicine, Beni-Suef University, Beni-Suef 62514, Egypt; mona.neuro@yahoo.com; 10 Department of Clinical and Chemical Pathology, Faculty of Medicine, Beni-Suef University, Beni-Suef 62514, Egypt; emanhamdy@med.bsu.edu.eg

**Keywords:** intensive care units, MDROs, infections, antibiotics, critically ill patients

## Abstract

*Background and Objectives:* This study aims to identify risk factors associated with MDRO infections and assess their impact on patient outcomes in Egyptian ICUs. *Materials and Methods*: The widespread overuse of antimicrobials has led to antibiotic multidrug resistance, posing significant challenges in intensive care units (ICUs) and leading to increased morbidity, mortality, and healthcare costs. A prospective observational study was conducted over 12 months, including 113 adult patients admitted to the ICU with confirmed bacterial infections. Comprehensive medical assessments and routine investigations were performed, including multisource cultures based on clinical suspicion. Patient histories, underlying conditions, and disease progression were documented. Patients were classified into two groups: those infected with MDROs and those without MDRO infections. *Results*: Significant differences were observed between patients with and without MDRO infections regarding temperature, pH, PaO_2_, HCO_3_, serum creatinine levels, high-dose inotropes, and inotrope dependence (*p*-values: 0.01, 0.028, 0.036, 0.008, <0.001, 0.013, 0.029, 0.039, <0.001, and 0.003, respectively). Additionally, cerebrovascular stroke and renal failure were significantly more frequent in MDRO-infected patients (*p*-values: 0.048 and 0.007, respectively). MDROs accounted for 42% of infections. The most commonly detected MDRO was *Klebsiella* spp. (52%). Patients with MDRO infections showed significantly higher mortality (42.6%), increased incidence of ARDS, invasive ventilation, and longer ventilation durations. Independent risk factors included prior antibiotic use (OR: 3.2; 95% CI: 1.5–6.8) and invasive device presence (OR: 2.7; 95% CI: 1.2–5.9). *Conclusions*: Cerebrovascular stroke and renal failure appear to be risk factors for MDRO infections. MDRO infections in ICUs are associated with poor clinical outcomes and increased complications. Improved antimicrobial stewardship and targeted prevention strategies are urgently required.

## 1. Introduction

Antimicrobial resistance remains one of the most pressing global health threats [1]. Critically ill patients in ICUs are particularly vulnerable due to frequent invasive procedures and broad-spectrum antibiotic use [2]. Globally, the post-COVID-19 era has seen a surge in MDRO prevalence, partially due to empirical antibiotic use during viral infections [3].

In fact, the genes responsible for drug resistance in certain bacterial strains predate antibiotics by millions of years [4]. However, the growing prevalence of multidrug resistance has become an increasingly serious challenge, leaving healthcare providers to combat a formidable enemy with limited resources [5].

Patients in intensive care units (ICUs) are particularly vulnerable to infections due to their critical conditions, which can weaken immune responses [6]. Additionally, their frequent exposure to invasive medical devices further increases the risk of infection [7]. 

Studies estimate that up to 50% of ICU patients receive empirical antibiotic therapy before the specific infecting organism is identified, significantly contributing to the spread of multidrug-resistant organisms (MDROs). Strong evidence suggests a direct correlation between antibiotic resistance rates and antibiotic consumption [8].

The Centers for Disease Control and Prevention (CDC) has identified the global increase in MDROs as a major therapeutic challenge that significantly affects patient outcomes in ICUs [9]. Consequently, infection prevention and management are crucial in reducing morbidity and mortality in these settings [10]. 

The emergence and proliferation of multidrug-resistant organisms (MDROs) in intensive care units (ICUs) represent a significant global health concern. ICUs are particularly susceptible due to the high usage of broad-spectrum antibiotics, invasive procedures, and the presence of immunocompromised patients [11]. Recent Egyptian data indicate carbapenem-resistant Klebsiella pneumoniae (CRKP) prevalence rates ranging from 14.2% to 66%, emphasizing the need for localized research [12]. This study aims to identify MDRO infection risk factors and outcomes to guide antimicrobial stewardship and infection control strategies.

## 2. Materials and Methods

### 2.1. Study Design and Setting

This prospective observational study was conducted on 113 culture-positive ICU patients diagnosed with infection either clinically or based on investigations. Patients were recruited from the General ICU, Surgical ICU, Chest ICU, and Tropical intermediate care unit between August 2022 and August 2023. Informed consent was obtained for each patient, and the study received ethical approval (FMBSUREC/02102022/Mohamed).

### 2.2. Inclusion and Exclusion Criteria

Inclusion: Adult patients (≥18 years) with culture-confirmed bacterial infections were included.

Exclusion

**Figure 1:**
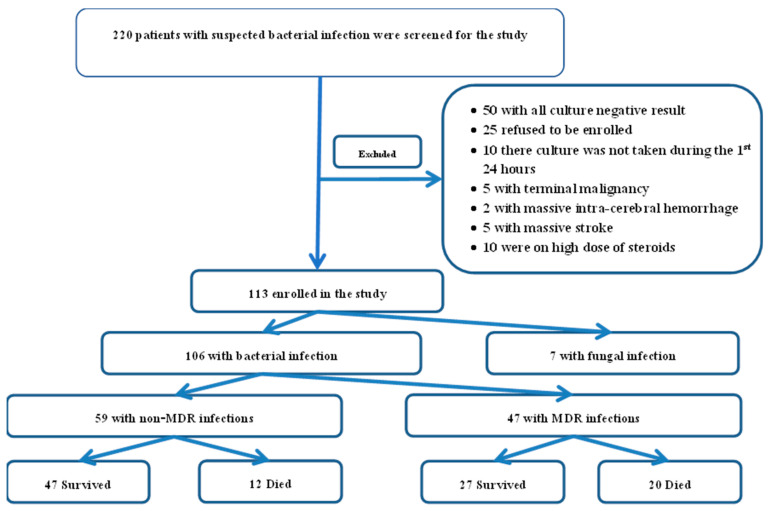
Flow diagram of the included and excluded patients.

Patients with irreversible brain damage, acute or chronic neuromuscular diseases; those on immunosuppressive therapy for any indication; individuals with terminal malignancies, end-stage idiopathic pulmonary fibrosis, end-stage organ failure, pregnant women; and culture-negative patients were excluded. A flow diagram of the included and excluded patients is demonstrated in Figure 1.

### 2.3. Definition of MDROs

MDROs were defined as microorganisms resistant to at least one agent in three or more antimicrobial classes, in accordance with CDC and ECDC criteria.

### 2.4. Data Collection

Data included demographics, comorbidities (e.g., diabetes mellitus, chronic kidney disease), prior antibiotic use within 90 days, invasive devices, labs, and clinical outcomes. ICU interventions and organ failure were documented using the qSOFA and Glasgow Coma Scale.

### 2.5. Clinical Assessment

The patients underwent comprehensive clinical assessments, including detailed examinations of the cardiac, respiratory, abdominal, and neurological examination. Consciousness level was evaluated using the Glasgow Coma Scale [13]. Additionally, organ failure was assessed using the quick Sequential Organ Failure Assessment score [14]. It was calculated based on laboratory and clinical data recorded within 48 h of ICU admission.

The following clinical variables were documented: admission temperature, heart rate, respiratory rate and oxygen saturation were noted, need for vasopressors (including dopamine, epinephrine, norepinephrine, and dobutamine) and their prescribed doses (low dose: less than half of the dose rang or high dose: higher than half of the dose rang), requirement and duration of mechanical ventilation (invasive and non-invasive), and patients’ ICU stay. Corrected durations were calculated to overcome the problem of mortality bias.

Infection vs. Colonization: Infection was defined by the presence of clinical signs and symptoms consistent with infection, accompanied by a positive culture from a sterile site. Colonization, identified by positive cultures without clinical signs of infection, was excluded from the analysis.

### 2.6. Infection Sites

Infections were categorized based on their sites: respiratory tract (sputum culture), urinary tract (urine culture), blood (blood culture), and others (intra-abdominal, soft tissue, pleural fluid, and acetic fluid).

### 2.7. Laboratory and Culture Techniques

Baseline laboratory data retrieved from patient files included inflammatory markers (C-reactive protein and erythrocyte sedimentation rate, when indicated), hemoglobin, hematocrit, white blood cells, platelets, creatinine, sodium (Na), potassium (K), AST, ALT, PH, PCO_2_, PaO_2_, HCO_3_, and cultures results (sputum, blood, urine, etc.) according to the clinical need.

Regarding cultures, samples were obtained within the first 24 h of ICU admission, and their standard operating procedures were established for all procedures, including sample collection, transport, registration, processing, and reporting. Kirby–Bauer disk diffusion and automated Vitek 2 (Biomerieux, Marcy-l’Étoile, France) systems were used. The results were interpreted per CLSI 2020 guidelines.

The Clinical Laboratory and Standards Institute standards [15] served as references for laboratory methods. The pathogen identification of isolates and antimicrobial susceptibility testing were performed using the automated Vitek 2 system (Biomerieux, Marcy-l’Étoile, France).

Outcomes: Patients were followed until discharge or death in the hospital for a maximum of 30 days from ICU admission.

### 2.8. Sample Size Calculation

The sample size was calculated using Epi Info v3.5.1. Based on a previous study (Rodríguez-Villodres et al., 2021) with an MDRO prevalence at 11.6% [16], a minimum of 111 patients was required to achieve a 95% confidence level and 5% margin of error.

### 2.9. Statistical Analysis

Data were analyzed using SPSS version 24.0 (IBM, Armonk, NY, USA). Continuous variables are expressed as mean ± SD or median (IQR), and categorical variables are reported as frequencies and percentages. Univariate analyses were performed using the chi-square test or Fisher’s exact test for categorical variables and a *t*-test or Mann–Whitney U test for continuous variables. Variables with *p* < 0.1 in univariate analysis were included in a multivariate logistic regression model to identify the independent risk factors for MDRO infections. False discovery rate (FDR) correction was applied for the key outcomes. A *p*-value < 0.05 was considered statistically significant.

## 3. Results

### 3.1. Patient Demographics and Baseline Characteristics

The mean age of the 113 patients enrolled was 53 ± 14.8 years; 56.6% were male. Of the 113 patients, 106 had bacterial infections (59 non-MDRO, 47 MDRO), and 7 had fungal infections (Figure 1).

Common comorbidities included diabetes mellitus, chronic kidney disease, and cerebrovascular accidents.

No significant differences were found between MDRO-infected and non-MDRO-infected patients regarding age or sex (*p* = 0.19 and 0.345, respectively) (Table 1).

### 3.2. Clinical and Laboratory Comparison Between MDRO and Non-MDRO Groups

After applying the Benjamini–Hochberg false discovery rate (FDR) correction, several differences remained statistically significant: temperature (*p* = 0.01), pH (*p* = 0.028), PaO_2_ (*p* = 0.036), HCO_3_ (*p* = 0.008), serum creatinine (*p* < 0.001), mortality (*p* = 0.013), ARDS incidence (*p* = 0.029), invasive ventilation use (*p* = 0.039), the need for inotropes (*p* = 0.003), and the use of high-dose inotropes (*p* < 0.001).

Other parameters (HR, RR, PCO_2_, hemoglobin, hematocrit value, TLC, PLT, the use of NIV, days on NIV, days on invasive ventilation, ICU stay, and hospital stay before admission to the ICU) showed no significant differences (Table 2).

### 3.3. Duration of Ventilation and ICU Stay

To address potential bias from early death in duration analysis, we conducted a sensitivity analysis using Kaplan–Meier survival curves, instead of assigning fixed 30-day durations.

- Days on NIV (Mean ± SD): non-MDRO: 8.4 ± 11.4 vs. MDRO: 14.8 ± 13.8 (*p* = 0.011);
- Days on invasive ventilation: non-MDRO: 6.4 ± 12.1 vs. MDRO: 12.8 ± 15 (*p* = 0.017).

Kaplan–Meier log-rank test confirmed prolonged ventilation in MDRO patients (*p* = 0.019). The total ICU stay remained non-significant (*p* = 0.086) (Table 3).

### 3.4. Comorbidities and Risk Factors

Significant comorbidities were more frequent in the MDRO group: cerebrovascular stroke (*p* = 0.048) and renal failure (*p* = 0.007). No significant differences were observed in diabetes (*p* = 0.69), hypertension (*p* = 0.98), heart failure, or IHD (Table 4).

### 3.5. MDRO Distribution by Site and Organism

Among the MDRO cases, *Klebsiella* spp. was the most common (52%), followed by *Acinetobacter* spp. (18%), *Pseudomonas* spp. (8%), and *E. coli* spp. (8%). The most frequent sites of isolation were sputum > blood > urine (Table 5).

### 3.6. Multivariate Logistic Regression Analysis

Independent predictors of MDRO infection were as follows:

- Prior antibiotic use within 90 days (OR: 3.2; 95% CI: 1.5–6.8; *p* = 0.003);
- Presence of invasive devices (OR: 2.7; 95% CI: 1.2–5.9; *p* = 0.01).

## 4. Discussion

This study evaluated 113 ICU patients with confirmed infections, finding that 42% were infected with MDROs, primarily *Klebsiella* spp. (52%), consistent with global concerns regarding carbapenem-resistant Enterobacteriaceae.

By analyzing regional data, we aimed to improve the local mapping of bacterial resistant organisms to enhance management strategies and predict infection outcomes for critically ill patients and the factors affecting them.

The post-COVID-19 environment has amplified MDRO challenges, with factors such as empirical antibiotic overuse and longer ICU stays contributing to rising resistance rates [17]. Our findings are aligned with global data, suggesting that 20–40% of ICU patients worldwide are affected by MDROs, though geographic and institutional variations are substantial [18]. 

Age, sex, heart rate, respiratory rate, admission systolic blood pressure, admission diastolic blood pressure, PCO_2_, hemoglobin, hematocrit value, TLC, platelet count, and hospital stay before admission to the ICU were similar between non-MDRO-infected patients and those with MDRO infections. Similarly, in another study [19], MDRO-infected patients had a similar age and gender distribution as controls. Also, groups spent a similar number of days in the hospital prior to infection. In contrast, another study [6] found that longer ICU stay prior to infection had higher rates of MDRO infections. This difference may be because none of our patients were admitted to the ICU, which may have reduced their exposure to MDROs [20]. 

Importantly, MDRO infections were associated with significantly worse clinical outcomes, including higher mortality (42.6%), greater need for inotropes, more frequent ARDS, and longer durations of mechanical ventilation. These outcomes underscore the clinical burden posed by MDROs in critical care.

Consistent with previous studies, serum creatinine was significantly higher in MDRO-infected patients, reflecting potential renal dysfunction as both a risk factor and consequence of infection. Additionally, arterial blood gas abnormalities (lower pH, PaO_2_, and HCO_3_) point to more severe physiological compromise in the MDRO group.

Regarding comorbidities, renal failure and cerebrovascular stroke were significantly associated with MDRO infection. These conditions may reflect both increased susceptibility and greater healthcare exposure [21]. Our findings are in agreement with those of Su et al. (2018) [22], who reported a strong link between chronic kidney disease and MDRO colonization/infection.

Other risks include broad-spectrum antibiotic use, malnutrition, invasive procedures, and infections from hemodialysis [23]. Patients with kidney disease have impaired immune systems, which persist despite treatments like dialysis [24]. Additionally, Alsehemi et al. noted significantly higher incidence of stroke (*p* = 0.046) and hemiplegia (*p* = 0.007) in MDRO-infected patients [25]. Heart failure, hypertension, and ischemic heart disease were similar in both groups, and unexpectedly, diabetes mellitus was not significantly associated with MDROs in our cohort, in contrast to multiple studies suggesting a higher MDRO risk in diabetics [26], particularly in diabetic foot infections [27]. In contrast, Trivedi et al. noted a non-significantly higher prevalence of MDROs in wounds of diabetic patients [28]. This discrepancy may stem from our study’s relatively low diabetes prevalence (35%) or differing patterns of antimicrobial use. Methodological differences, including study design and infection type, may also account for this variation [29]. Most of the MDROs in our study were Gram-negative, similar to some studies that showed that Gram-negative bacteria outnumber Gram-positive bacteria in patients with numerous injuries [25]. Cohen et al. [30] found that mortality rates were significantly higher in Gram-negative bacteremia, whereas Zahar et al. [31] did not find this correlation.

Our study found that Klebsiella species was the leading MDR pathogen in ICU patients, notably in sputum, blood, and urine cultures. This aligns with global concerns about carbapenem-resistant Klebsiella pneumoniae. It was followed by *Acetobacter* spp. Pseudomonas and *E. Coli* spp., and *Enterobacter* spp. were also present, especially in respiratory and urinary infections. Similar findings were reported worldwide [32]. Another study noted that the most common were Klebsiella species (27%), *Escherichia coli* (25%), Pseudomonas species (24%), and Acinetobacter species (17%) [33]. Also, others noted similar results [25]. In contrast, an earlier study showed that *Enterococcus* spp. (10.8%), Staphylococcus aureus (47.3%), and *Candida* spp. (10.1%) were the most common infections [34,35]. Also, in another study, *E. coli* was the most common organism (39.4%), followed by *S. aureus* (25.6%) and *P. aeruginosa* (18.2%) [36]. Another study noted that the predominant organisms were *S. aureus* (despite the overall predominance of Gram-negative organisms) [37]. Interestingly, Acinetobacter was involved in 9% of all infections, similar to the rate reported in the first EPIC study [38] but considerably higher than the 3.6% reported in the more recent SOAP study [37]. In another study, Acetobacter was notably present in sputum (16%) and urine (40%) cultures [39]. These variations are mostly due to differences in the studied population, location, and antimicrobial strategies in different regions of the world [40]. Regarding days on NIV, days on invasive ventilation, and ICU stay, they were similar in both groups (*p* = 0.55, 0.9, 0.36, respectively) but after adjustment of the stay of deceased patients to 30 days, we found significant difference in both days on NIV and days on invasive ventilation (*p* = 0.011 and 0.017, respectively), while total ICU stay remained similar in both groups (*p* = 0.086). The use of invasive ventilation was also significantly high in MDRO-infected patients (*p* = 0.039). Similarly, Magiraa et al. noted higher median days on ventilation after MDRO infection, which was not significant (*p* = 0.054). In contrast, they noted that MDRO-positive patients spent a greater median number of total days in hospital after infection (*p* = 0.005, OR = 1.04) [19]. There are some studies that showed little or no effect of multidrug resistance on the hospital stay [41]. It seems that comorbidities are the main determinants of prolonged hospital stay [42].

We observed a higher need for vasopressors and higher-dose inotrope use among MDRO patients, highlighting the role of these infections in hemodynamic instability and sepsis severity. Alkofide et al. found that MDRO infections often cause hemodynamic instability, necessitating more inotropes [43].

This supports the Surviving Sepsis Campaign’s emphasis on early, appropriate antimicrobial therapy [44]. 

The authors of [45] revealed that the association between MDROs and increased ARDS incidence (*p* = 0.029) and mortality (*p* = 0.013) may be related to the higher virulence of the organisms or the delayed initiation of effective antimicrobial therapy. This is supported by several other studies [46,47]. Despite the use of multivariate regression, residual confounding may exist. Variables such as colonization status prior to ICU admission, duration of antibiotic exposure, and environmental contamination were not controlled for.

We note that only clinically significant infections from sterile sites were included. Patients with colonization but no clinical symptoms were excluded, although the lack of molecular typing is a limitation in distinguishing strain-specific dynamics [48]. 

In contrast to studies that found no mortality differences [49], our data suggest that MDRO infections contribute directly to poor outcomes. However, further multicenter prospective studies with genetic typing are needed to confirm causality and explore transmission patterns.

The high prevalence of MDRO infections, particularly CRKP, in our ICU aligns with national data indicating variable but concerning rates of carbapenem resistance. The identification of prior antibiotic use and invasive devices as risk factors underscores the need for stringent antimicrobial stewardship and infection control practices [50]. 

The association between higher SOFA scores and MDRO infections suggests that patients with greater severity of illness are more susceptible, highlighting the importance of early identification and targeted interventions.

Our findings emphasize the necessity for localized surveillance data to inform empirical antibiotic therapy and guide infection prevention strategies.

### Limitations

This study’s observational design and single-center setting may limit the generalizability of the findings. The absence of molecular typing precluded the identification of specific resistance genes. Additionally, the lack of correction for multiple comparisons in statistical analyses may increase the risk of Type I errors.

## 5. Conclusions

Our study highlights the high prevalence of multidrug-resistant organism (MDRO) infections in Egyptian intensive care units, with *Klebsiella* spp. (particularly carbapenem-resistant strains) emerging as the predominant pathogen. These infections were associated with significantly worse clinical outcomes, including increased mortality, higher incidence of ARDS, and prolonged need for mechanical ventilation and inotropic support. Independent risk factors such as prior antibiotic use within 90 days and the presence of invasive medical devices underscore the importance of strengthening antimicrobial stewardship programs and implementing rigorous infection prevention strategies.

While our findings support the urgency for localized interventions, we caution against generalizing these results due to the study’s single-center setting and lack of molecular resistance profiling. Thus, multicenter surveillance and genomic analysis are necessary to further understand resistance patterns and transmission dynamics.

Future directions should include integrating routine resistance monitoring, enhancing diagnostic capabilities, and fostering inter-hospital collaboration to curb the spread of MDROs and improve patient outcomes in critical care settings.

## Figures and Tables

**Table 1:** Demographic data of the included patients.

		Non-MDRO-Infected Patients (n = 59)	MDRO-Infected Patients (n = 47)	*p*-Value
Age (Mean ± SD)		55 ± 12.8	50.6 ± 17	0.19
Sex	Male [n (%)]	31 (52.54%)	28 (59.57%)	0.345
	Female [n (%)]	28 (47.46%)	19 (40.43%)	

MDRO: multidrug-resistant organism (s); *p*-value > 0.05 is considered non-significant.

**Table 2:** Comparison between non-MDRO-infected and MDRO-infected patients in clinical and laboratory parameters.

Parameters	Non-MDRO-Infected Patients (n = 59)	MDRO-Infected Patients (n = 47)	*p*-Value
Temperature (°C) (Mean ± SD)	37.7 ± 0.6	38.0 ± 0.5	0.01 *
HR (Beats/min) (Mean ± SD)	106 ± 19	106 ± 13	0.92
RR (Breaths/min) (Mean ± SD)	20 ± 7	22.7 ± 6	0.064
Admission SBP (mmHg) (Mean ± SD)	105 ± 21	102 ± 34	0.64
Admission DBP (mmHg) (Mean ± SD)	64 ± 13	65 ± 21	0.78
PH (Mean ± SD)	7.38 ± 0.09	7.33 ± 0.11	0.028 *
PCO_2_ (mmHg) (Mean ± SD)	40.8 ± 16	35 ± 10.3	0.074
PaO_2_ (mmHg) (Mean ± SD)	60 ± 26	71 ± 29.3	0.036 *
HCO_3_ (mmol/dL) (Mean ± SD)	24.8 ± 9.5	20.5 ± 6.85	0.008 *
Hemoglobin (g/dL) (Mean ± SD)	9.9 ± 1.6	9.4 ± 1.8	0.176
Hematocrit value (%) (Mean ± SD)	29.5 ± 5.5	27.9 ± 5.1	0.134
TLC (10^3^ cell/mm^3^) (Mean ± SD)	14.4 ± 7.7	16.8 ± 8.22	0.12
Platelets (10^3^/mm^3^) (Mean ± SD)	203 ± 142	166 ± 124	0.168
Serum creatinine (mg/dL) (Mean ± SD)	1.41 ± 0.7	2.34 ± 1.8	<0.001 *
Non-survivors [n (%)]	12 (20.3%)	20 (42.6%)	0.013 *
ARDS [n (%)]	4 (6.8%)	10 (21.3%)	0.029 *
Use of invasive ventilation [n (%)]	14 (23.7%)	20 (42.6%)	0.039 *
Use of NIV [n (%)]	23 (39%)	17 (36.2%)	0.767
Use of high doses of inotropes [n (%)]	2 (3.4%)	13 (27.7%)	<0.001 *
Need for inotropes [n (%)]	16 (27.1%)	26 (55.3%)	0.003 *
Days on NIV (Mean ± SD)	3.27 ± 5	2.68 ± 5	0.55
Days on invasive Ventilation (Mean ± SD)	1.95 ± 4.9	2 ± 5	0.91
ICU stay (days) (Mean ± SD)	13 ± 9.9	11.6 ± 5.9	0.36
Hospital stay before ICU (Mean ± SD)	1.2 ± 2.4	1.28 ± 3.7	0.9

ARDS: acute respiratory distress syndrome, DBP: diastolic blood pressure, HR: heart rate, ICU: intensive care unit, MDRO: multidrug-resistant organism (s), NIV: non-invasive ventilation, PaO_2_: partial pressure of arterial oxygen, PCO_2_: partial pressure of carbon dioxide, RR: respiratory rate, SBP: systolic blood pressure, TLC: total leukocyte count; * *p*-value ≤ 0.05 is considered significant.

**Table 3:** Comparison between non-MDRO-infected and MDRO-infected patients in corrected duration analysis.

Parameters	Non-MDRO-Infected Patients (n = 59)	MDRO-Infected Patients (n = 47)	*p*-Value
Days on NIV (Mean ± SD)	8.42 ± 11.4	14.79 ± 13.8	0.011 *
Days on invasive ventilation (Mean ± SD)	6.42 ± 12.1	12.79 ± 15	0.017 *
ICU stay (days) (Mean ± SD)	15.88 ± 11	19.5 ± 10.2	0.086

ICU: intensive care unit, MDRO: multidrug-resistant organism (s), NIV: non-invasive ventilation; * *p*-value ≤ 0.05 is considered significant.

**Table 4:** Comparison between non-MDRO-infected and MDRO-infected patients in comorbidities.

Comorbidities	Non-MDRO-Infected Patients (n = 59)	MDRO-Infected Patients (n = 47)	*p*-Value
Cerebrovascular stroke [n (%)]	1 (1.7%)	5 (10.6%)	0.048 *
Hypertension [n (%)]	25 (42.4%)	20 (42.6%)	0.98
Diabetes mellitus [n (%)]	21 (35.6%)	15 (31.9%)	0.69
Renal failure [n (%)]	9 (15.3%)	18 (38.3%)	0.007 *
Heart Failure [n (%)]	3 (5.1%)	2 (4.3%)	0.84
Ischemic heart disease [n (%)]	1 (1.7%)	2 (4.3%)	0.43

MDRO: multidrug-resistant organism (s); * *p*-value ≤ 0.05 is considered significant.

**Table 5:** MDROs prevalence according to the culture site.

Organism	Sputum (n = 25)	Blood (n = 15)	Urine (n = 10)	Total (n = 50)
*Klebsiella* spp.	15 (60%)	7 (46.7%)	4 (40%)	26 (52%)
*Pseudomonas* spp.	2 (8%)	2 (13.3%)	-	4 (8%)
*Acetobacter* spp.	4 (16%)	1 (6.7%)	4 (40%)	9 (18%)
*E. coli* spp.	1 (4%)	2 (13.3%)	1 (10%)	4 (8%)
*Enterobacter* spp.	-	3 (20%)	1 (10%)	4 (8%)
Pseudomonas and Klebsiella	2 (8%)	-	-	2 (4%)
Acinetobacter–Klebsiella	1 (4%)	-	-	1 (2%)

spp.: species.

## Data Availability

The datasets generated during and/or analyzed during the current study are available from the corresponding authors upon reasonable request.
